# Effects of oregano essential oil on the ruminal pH and microbial population of sheep

**DOI:** 10.1371/journal.pone.0217054

**Published:** 2019-05-20

**Authors:** Rui Zhou, Jianping Wu, Liping Zhang, Lishan Liu, David P. Casper, Ting Jiao, Ting Liu, Jianfu Wang, Xia Lang, Shuzhen Song, Xuyin Gong

**Affiliations:** 1 College of Animal Science and Technology, Gansu Agricultural University, Lanzhou, Gansu, People’s Republic of China; 2 Animal Husbandry, Pasture, and Green Agriculture Institute, Gansu Academy of Agricultural Sciences, Gansu, People’s Republic of China; 3 Furst-McNess Company, Freeport, IL, United States of America; 4 College of Grassland Science, Key Laboratory of Grassland Ecosystem, Gansu Agricultural University, Lanzhou, People’s Republic of China; University of Illinois, UNITED STATES

## Abstract

Oregano essential oil (OEO), which has antimicrobial properties, may be used for altering the ruminal pH and microbial populations of sheep, as observed by the altered volatile fatty acid patterns. To further elucidate the effects of OEO on ruminal pH and microbial populations of sheep, 3 German merino sheep × local sheep crossbred rams with permanent ruminal fistulas were randomly assigned to a 3 × 3 Latin square design with 12-d periods. The treatments were as follows: control (CON); OEO4: OEO supplied at 4 g•d^-1^; and OEO7: OEO supplied at 7 g•d^-1^. Starting on day 11, rumen fluid was collected at 0 h, and at 4, 8, 12, 24 and 48 h after supplying OEO, and then pH values of rumen fluid were immediately measured. The abundance of microbial populations was determined by using qPCR. The ruminal pH values were similar among the sheep from all treatments. The abundance of ruminal fungi was higher for the sheep supplied OEO7 compared with the sheep supplied CON and OEO4, especially at 4 and 12 h. The abundance of ruminal protozoa decreased with supplied OEO, indicating that OEO could inhibit the protozoa. The abundance of the total ruminal bacteria was similar for the sheep from all treatments, but *R*. *flavefaciens*, *R*. *albus* and *F*. *succinogenes* increased in the sheep supplied OEO4 compared with those in the sheep supplied CON, however, the sheep supplied OEO7 had higher abundances of *R*. *flavefaciens* than the sheep supplied CON. These results demonstrated that supplying OEO to sheep did not affect the ruminal pH but could shift the rumen microbial population to one with less protozoa. Supplying OEO can preferentially enhance the growth of certain rumen microbial populations, but the shifts were influenced by the supply rate. Therefore, supplying low amount (i.e. 4 g•d^-1^) of OEO could have positive effects on ruminal microbial populations, whereas supplying elevated doses of OEO could be detrimental to those same ruminal microbial populations.

## Introduction

In recent decades, a large number of chemical additives (antibiotics, ionophores, etc) have been used in ruminant production to modulate rumen fermentation and to improve growth and feed efficiency. However, the residues of most chemical additives in animal products and bacterial resistence to antibiotics led to the growing public concern over the health risks and environment impacts [1, 2]. As a result, a number of studies have been conducted to investigate natural alternatives (essential oils, tannins, saponins, etc) to the chemical feed additives in ruminant production. Among these natural alternatives, essential oils (EOs) have been demonstrated that could manipulate rumen fermentation [3, 4], alter bacterial growth and metabolism of several rumen bacteria [5, 6], and improve ruminant performance [4].

Oregano essential oil (OEO) is an aromatic volatile oil extracted from oregano (*Origanum vulgare L*.). The principal compounds of OEO are carvacrol, thymol, γ-terpinene, *p*-cimene and linalool [7]. Several studies have reported that OEO possesses antimicrobial [8, 9] and antioxidant properties [10, 11]. Feed efficiency was improved when feeding OEO via stimulating the beneficial gut microbes [12] while inhibiting pathogenic microorganism growth, i.e. *Escherichia coli* and *Staphylococcus aureus* [13]. Feeding OEO to swamp buffalos [14] and rabbits [15] reduced the total microbial load and specific pathogens. In the previous study, we observed that the NDF degradability was increased by supplying 4 g•d^-1^ OEO in the diet of sheep, and the concentrations of acetate and propionate were increased by supplying 7 g•d^-1^ OEO [16]. However, there is a lack of ruminant (sheep) data for hypothesis testing that feeding OEO maybe beneficial for altering ruminal pH and microbial populations. Therefore, the objective of the present study was to evaluate the effects of OEO on the ruminal pH and microbiota using qPCR in further.

## Materials and methods

### Animal and treatments description

All animal handling protocols in this study were approved by the Gansu Agricultural University Animal Care and Use Committee guidelines (approved ID: 2012-2-159) in compliance with the Regulations for the Administration of Affairs Concerning Experimental Animals (The State Science and Technology Commission of P. R. China, 1988).

Three German merino sheep × local sheep crossbred rams (average initial live body weigh 53.68 ± 2.14 kg) fitted with permanent ruminal fistulas were randomly assigned to a 3 × 3 Latin square design with three 12-d periods. The 3 treatments sheep fed a control ration (Table 1) without supplying OEO (CON), sheep supplied 4 g•d^-1^ of OEO (OEO4), and sheep supplied 7 g•d^-1^ of OEO (OEO7). The OEO was supplied as a dry granular product (Ralco Inc., Marshall, MN, USA) containing 1.3% oregano essential oil from *Origanum vulgare* subsp.*hirtum* plants and 98.7% natural feed grade inert carrier. The OEO was supplied on the 11th day before feeding in the morning. The daily OEO dosages were accurately weighed and sealed in a 132 mm × 190 mm facial tissue (Hengan group co. LTD., Quanzhou, China) and then placed in the rumen through the rumen fistula and pushed below the rumen mat.

**Table 1:** Ingredient composition and nutrient concentrations of the diet.

**Ingredient, % of DM**^†^	
Corn	25.74
Cottonseed meal	2.34
Soybean meal	2.34
Rapeseed meal	2.34
Premix^†^, 1%	0.58
Limestone	0.58
Salt	0.58
Corn silage	65.5
**Nutrient, % of DM**	
Digestible energy, MJ•kg^-1^	18.39
Crude protein	18.13
Neutral detergent fiber	58.12
Acid detergent fiber	38.23
Calcium	0.41
Phosphorus	0.27

^†^The premix composition was as follows: vitamin A 630,000 IU; vitamin D 164,000 IU;vitamin E 1,260 IU;Fe 5,250 mg; Cu 2,100 mg; Mn 3,150 mg; Zn 5,250 mg; Se 32 mg; I 42 mg; and Co 63 mg per kg.

### Diets and management

Each experiment period lasted 12 d, which divided into the first 10 d for the rumen dietary adaptation to the treatment and the last 2 d for the sample collection. The sheep were housed in individual pens and fed twice daily at 07:00 and 19:00 h. Corn silage was the forage source and the dietary forage to concentrate ratio was 65.5:34.5. The ingredient composition of the diet (Table 1) was formulated to meet or exceed the NRC (2007) nutrient requirements of sheep [17]. All sheep had *ad libitum* access to feed and water.

### Ruminal fluid collection and processing

The ruminal fluid was evacuated manually through the rumen fistula before adding OEO at 0 h and additional rumen fluid samples were collected at 4, 8, 12, 24, and 48 h after supplying. When the rumen fluid was evacuated from the rumen, the initial 10 ml was discarded, and the subsequent 50 ml collected was used for the determination of pH and the microorganisms analyses. The collected rumen fluid was squeezed through 4 layers of 100 mm × 100 mm medical gauze (Winner Inc., LTD, Shenzhen, China). The pH value of the ruminal fluid was immediately measured and recorded using a glass electrode pH meter (Type CG 842, Blueline 14 pH, Schott Instruments, Deutschland, Germany) and the samples were immediately frozen and stored at -80°C (ULT Freezer Model DW-86L828, Haier Biomedical, Zingdoo, China) until DNA extraction.

### Rumen microbial DNA extraction

The DNA extraction procedures were performed by using a super clean bench (SW-CJ-2FD, Airtech, Ltd., Suzhou, China) to ensure that the samples were protected from environmental contaminations. The microbial DNA was extracted from the ruminal fluid samples using a TIANamp Stool DNA Kit (Tiangen Biotech Co., LTD., Beijing, China) according to the manufacturer’s instructions with slight modifications. Briefly, 1 ml of the frozen rumen fluid samples was centrifuged (TGL-16, Cence, LTD., Changsha, China) at 800 × g for 5 min at 4°C after thawing. The supernatant of each sample was collected and then centrifuged at 12000 × g for 30 min at 4°C. The sediments of the second centrifuged samples were transferred to 2 ml tubes for cell lysis by subjecting the samples twice to a 5 min pulse using the Mini-Beadbeater-8TM (BioSpec Products, Inc., Bartlesville, OK, USA) with 1 min cooling in ice between the two pulses. The DNA yield and purity were determined using a spectrophotometer (NanoPhotometer Pearl 360 Thermo Scientific, Implen, Germany). The DNA samples were stored at -20°C until further analysis.

### Design and synthesis of the PCR primers

The primers designed for detection of the targeted species were given in Table 2. The 16S rRNA sequences of *F*. *succinogenes* and *R*. *ablus*, and the 18S rRNA sequences of the fungi and protozoa were downloaded from GenBank (National Center for Biotechnology Information, Bethesda, MD, USA). The sequence specific regions for a given species (with > 97% similarity) were designed online against the GenBank sequences with Primer Premier 3.0 (http://bioinfo.ut.ee/primer3-0.4.0/primer3/) to ensure primer specificity. All primers were tested for the requirements imposed by quantitative real—time PCR. The housekeeping gene was tested, and no significant effects were found (*P* > 0.05). The oligonucleotides were synthesized by Sangon Biological Engineering (Shanghai, China).

**Table 2:** Primer sequences for the qPCR of rumen microorganisms.

Item	Forward/reverse (F/R) primer sequences (5’-3’)	Amplification size/bp	GenBank number
Fungi	F: TGACTCAACACGGGGAAACT R: CCAACTAAGAACGGCCATGC	105	JX240418.1
Protozoa	F: TGACTCAACACGGGGAAACT R: TCCACCAACTAAGAACGGCC	109	AJ810076.1
Bacteria	F: CCTACGGGAGGCAGCAG R: ATTACCGCGGCTGCTGG	181	[18]
*R*. *flavefaciens*^†^	F: TCT GGA AAC GGA TGG TA R:CCTTTAAGACAGGAGTTTACAA	259	[19]
*R*. *ablus*^‡^	F: ATGCCGCGGTGAATACGTT R: TTCGACTGCTTCCTCCTTGC	107	X85098.1
*F*. *succinogenes*^§^	F: GATGAGCTTGCGTCCGATT R: ATTCCCTACTGCTGCCTCC	139	EU606019.1

^†^R. flavefaciens = Ruminococcus flavefaciens

^‡^R. albus = Ruminococcus albus

^§^F. succinogenes = Fibrobacter succinogenes

### Quantitative real-time PCR

The abundance of the protozoa was quantified as described by Sylvester et al. [20]. The abundance of the fungi and the target bacterial species were quantified using SYBR Green-based quantitative real-time PCR (qPCR) using the LightCycler 480 Real-Time PCR System (Roche Applied Science, Switzerland). A sample-derived qPCR standard was prepared using the respective specific PCR primer set and a composite DNA sample that was prepared by pooling an equal amount of all the metagenomic DNA samples [21]. The efficiency of the PCR amplification was checked for the various primer concentrations and annealing temperatures. The optimal amplification conditions for each primer pair were achieved with 0.36 μM of each primer, and the annealing temperature of each target microorganism was 60°C. The reaction mixture had a final volume of 20 μl, containing 10 μl of FastStart Essential DNA Green Master Mix (Roche, Germany), 2 μl (20 ng) of template DNA, 6.8 μl of nuclease-free water and 0.36 μM of each forward and reverse primer, respectively. The amplification program was as follows: an initial denaturation at 95°C for 10 min, followed by 40 cycles of denaturation at 95°C for 10 s, annealing at 60°C for 10 s, and a final elongation at 72°C for 15 s. The negative controls (without DNA template) were run with every assay to assess the overall specificity. The detection of the fluorescent product was set at the last step of each cycle. To determine the specificity of the amplification, an analysis of the product melting was performed after each amplification. The melting curve was obtained by slow heating with a 0.1°C•s^-1^ increment from 65°C to 95°C, with fluorescence collection at 0.1°C intervals. Each of the standards was purified using a PCR Purification kit (Qiagen, USA). For each of the standards, the 16S rRNA (or 18S rRNA) gene copy-number concentration was calculated based on the length of the PCR product and its mass concentration [21]. Tenfold serial dilutions were made in Tris-EDTA (TE) buffer prior to the qPCR assays. To minimize variations, the qPCR assay for each species or group was performed in triplicate for both the standards and the metagenomic DNA samples using the same master mix and the same PCR plate. The absolute abundance was expressed as the 16S rRNA (or 18S rRNA) gene copies/ml of each sample [6].

### Statistical analyses

All data were subjected to least squares ANOVA for a 3 × 3 Latin square design [22] by using the PROC MIXED procedure of SAS (version 9.4, SAS Institute Inc., Cary, NC). The linear statistical model used was:

$$Y_{ijkl}=\mu +T_{\iota }+S_{j}+P_{k}+C_{ijk}+H_{l}+T_{i}xH_{l}+e_{ijkl}$$

Where Y_ijkl_ was the dependent variable; μ was the overall mean; T_i_ was the treatment effect; S_j_ was the sheep effect, P_k_ was the period effect; C_ijk_ was the whole plot error; H_l_ was the sampling hour; T_i_ x H_l_ was the interaction of treatment effect by hour; and e_ijkl_ was the residual random error. Sampling hour was considered a repeated measurement in time having an autoregressive covariance structure. The period effect was found to be nonsignificant (*P* > 0.05). Whenever significant differences attributed to the treatment were detected, the Fisher’s LSD test [22] was used to separate the least squares treatment means. Significance was declared at *P* < 0.05 and trends were declared at 0.05 < *P* < 0.10.

## Results and discussion

**Fig 1:**
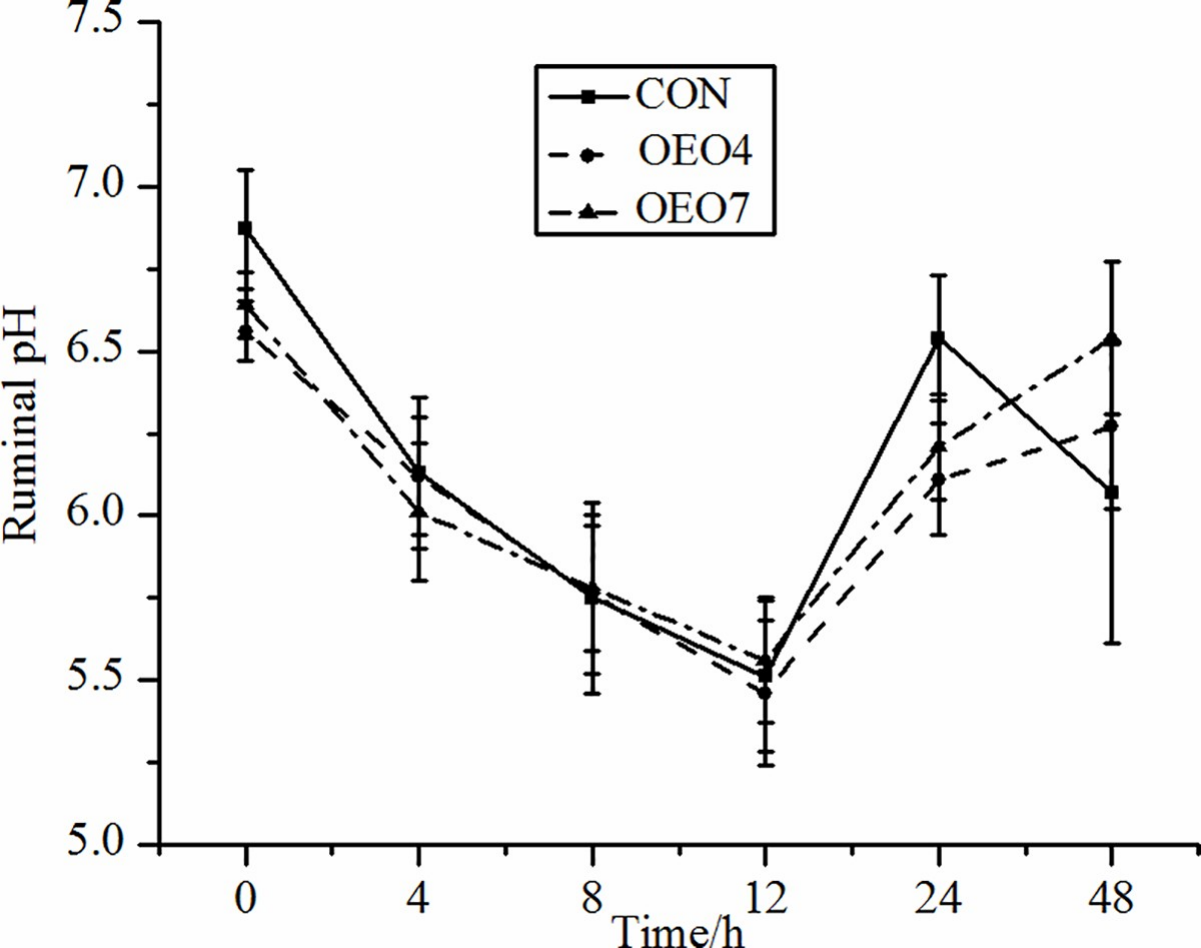
Ruminal pH for sheep fed oregano essential oil (OEO) at 0 (CON), 4 (OEO4), or 7 (OEO7) g•d^-1^. ^a, b, c^ Means within the same collection time with unlike superscripts differ, (*P* < 0.05).

It is known that the pH is an important index for evaluating the comprehensive EO effects for potentially altering the ruminal microbial population and fermentation to influence the ruminal environment [23]. In the present study, supplementing OEO to sheep resulted in no differences (*P* > 0.10) in the mean ruminal pH values compared with sheep supplied the CON ration (Table 3). In addition, the ruminal pH values were the highest before the OEO was supplied in the morning and declined afterward, as expected (Fig 1), with the lowest pH values being achieved at approximately 8 to 12 h after supplying OEO, but the sheep in all treatments were similar (*P* > 0.10) in their ruminal pH over the sampling times. These results corroborated the results from previous studies that showed that oregano leaf (essential oil presented 1.4% or 1.58% of oregano leaf DM) supplementation to lactating dairy cows had no effect on rumen pH [24, 25], while Yang et al. reported that adding a blend of garlic (5 g•d^-1^) and juniper berry (2 g•d^-1^) EO resulted in no effects on the ruminal pH value [26]. Meanwhile, Thao et al. reported that rumen pH in swamp buffaloes was unaffected by supplementing with eucalyptus EO (2 ml•hd^-1^•d^-1^) [14]. Lin et al. concluded that the ruminal pH value was not altered by the addition of a blend of EO (1.0 g•d^-1^) in the diet of Hu sheep [27]. Chaves et al. who reported that no change in the ruminal pH was detected when growing lambs were supplemented with cinnamon leaf, garlic, and juniper berry EOs (250 mg•L^-1^, respectively) [28].

**Table 3:** Effects of the different doses of OEO on the ruminal pH and the abundance of the ruminal microorganisms (copies number of 16S rRNA or 18S rRNA gene / ml).

Item	CON^†^	OEO4^‡^	OEO7^§^	*P*-value
pH	6.15±0.12	6.05±0.06	6.12±0.25	0.997
Fungi (× 10^4^)	2.27^b^±0.06	2.44^a^^b^±0.11	3.51^a^±0.18	0.038
Protozoa (× 10^4^)	5.77^a^±0.10	4.53^b^±0.10	4.59^b^±0.03	0.041
Bacteria (× 10^10^)	4.56^b^±0.11	4.91^a^±0.11	4.42^b^±0.10	0.080
*R*. *flavefaciens* (× 10^6^)	2.81^b^±0.10	3.64^a^±0.08	3.09^c^±0.04	0.001
*R*. *albus* (× 10^6^)	6.15^b^±0.05	7.57^a^±0.09	6.47^b^±0.11	<0.001
*F*. *succinogenes* (× 10^7^)	2.71^b^±0.04	3.30^a^±0.04	2.46^a^^b^±0.18	0.005

^a, b, c^ Means within a row with unlike superscripts differ, (*P* < 0.05).

^**†**^CON = supplied with no oregano essential oil.

^**‡**^OEO4 = supplied oregano essential oil at 4 g•d^-1^.

^**§**^OEO7 = supplied oregano essential oil at 7 g•d^-1^.

**Fig 2:**
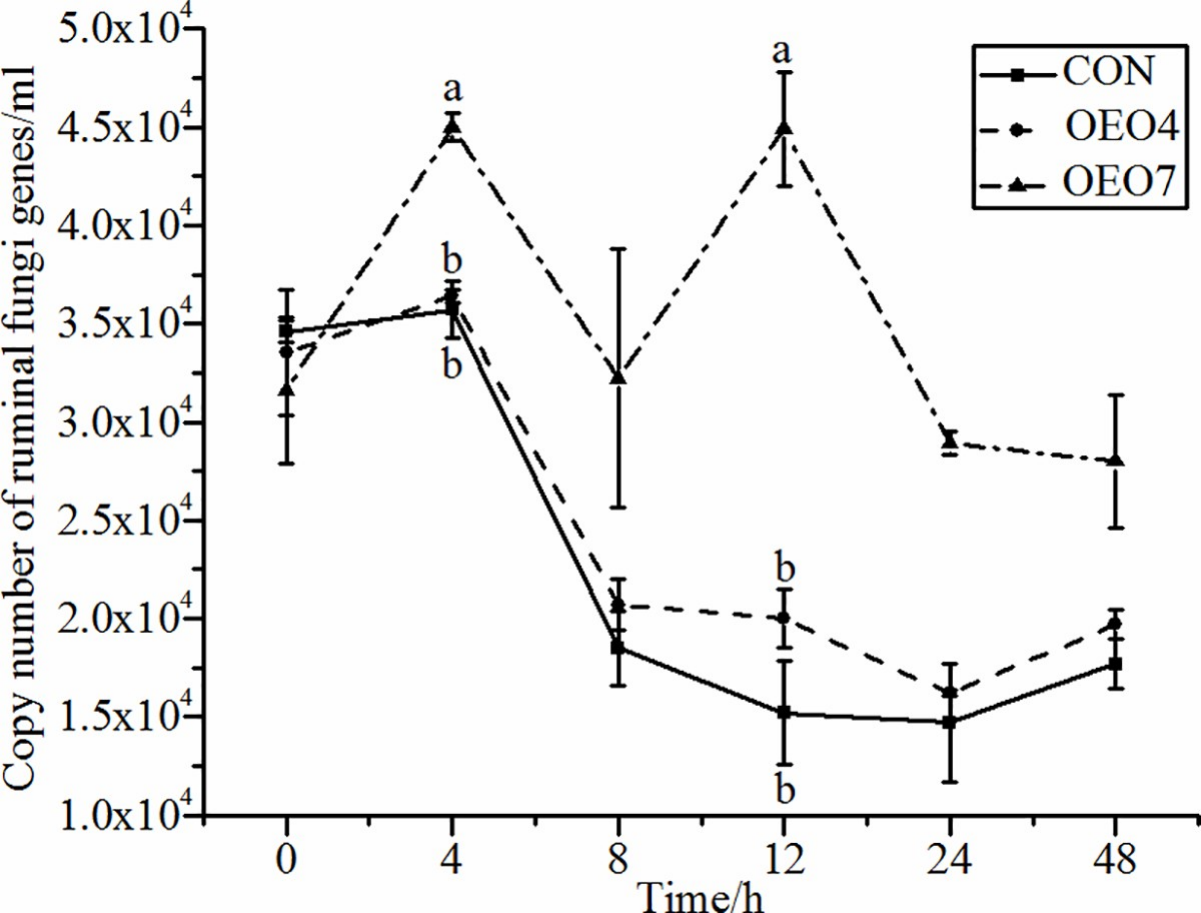
Abundance of ruminal Fungi for sheep fed oregano essential oil (OEO) at 0 (CON), 4 (OEO4), or 7 (OEO7) g•d^-1^. ^a, b, c^ Means within the same collection time with unlike superscripts differ, (*P* < 0.05).

Ruminal microbial fermentation is crucial for the optimal growth and meat or milk production of ruminants. Thus, the microbial composition and their functions as well as other factors (i.e., ration) affecting the rumen microbiome would all impact the nutrients available for absorption. During ruminal fermentation, it is well known that fungi can penetrate the cell wall to enhance cellulose degradation from lignin, which cannot be degraded by bacteria and protozoa [29]. These data demonstrate that sheep supplied OEO7 had greater (*P* < 0.05) abundances of fungi than the sheep supplied with CON or OEO4 (Table 3). Thus, feeding sheep with OEO at 7 g•d^-1^ shifted the microbial populations to enhance the proliferation of rumen fungi, which could lead to an enhancement in ruminal fiber digestion. However, Wang et al. reported there was a depression in fiber digestibility when sheep were supplied with 7 g•d^-1^ OEO. Therefore, the correlation between fungi and fiber digestibility needed to explore in depth through more studies [16]. In agreement with our results, Agarwal et al. reported that the ruminal fungi populations were increased four times and the *in vitro* true digestibility of feed was depressed when feeding a low amount (0.33 ml•L^-1^) of peppermint oil, and feeding a high amount (1.0 ml•L^-1^) decreased the ruminal fungi populations in an *in vitro* experiment [30]. In contrast, Lin et al. reported that supplementing with a blend of EO (clove, oregano, cinnamon, and lemon) did not affect the ruminal fungi populations [31]. Thus, the specific type of EO supplemented may explain the discrepancy between this study and the literature reporting fungi populations because not all EO would have the same antimicrobial properties. The sheep supplied OEO7 demonstrated an increase (*P* < 0.05) in the abundance of ruminal fungi at 4 and 12 h after feeding compared with sheep supplied CON and OEO4 (Fig 2). This finding agreed with the results of Denman et al., who reported that anaerobic fungi growth increased quickly between 4–8 h after culturing, and that a second growth spike appeared at 12 h [32]. Moreover, McIntosh et al. reported that feeding an EO mixture (thymol:eugenol:vanillin:limonene = 1:1:1:1) at levels greater than 20 mg•L^-1^ significantly inhibited rumen fungi growth [33]. These data suggest that the EO supplementation amount was an important factor affecting the ruminal microbial populations. However, to date, few studies have reported EO effects on rumen fungi. Therefore, further studies are required to elucidate the regulatory mechanisms of EO to enhance or inhibit rumen fungi.

**Fig 3:**
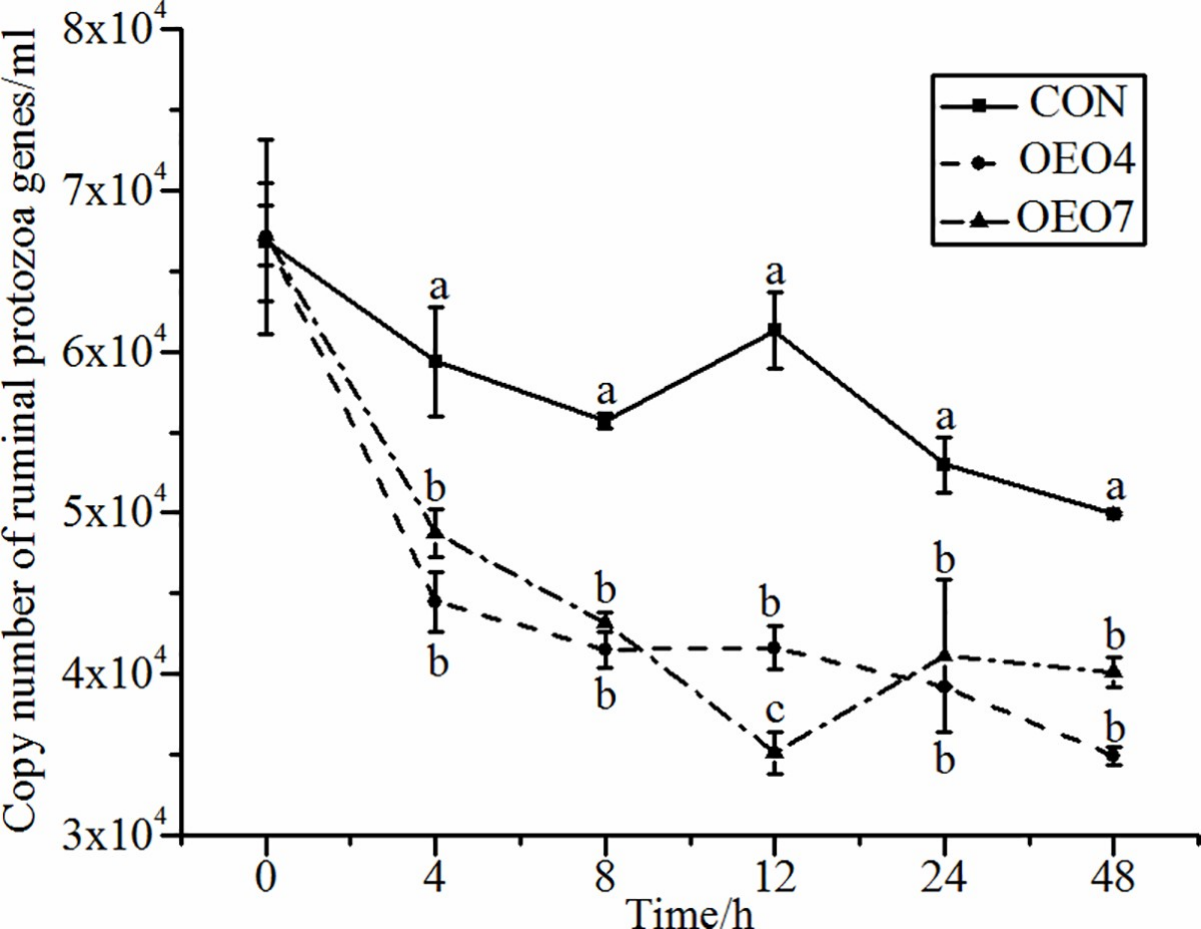
Abundance of ruminal Protozoa for sheep fed oregano essential oil (OEO) at 0 (CON), 4 (OEO4), or 7 (OEO7) g•d^-1^. ^a, b, c^ Means within the same collection time with unlike superscripts differ, (*P* < 0.05).

Protozoa, representing up to approximately 50% of the ruminal biomass, can make important contributions to nutrient degradation and the maintenance of the bacterial populations [20, 34]. The abundance of ruminal protozoa decreased significantly (*P* < 0.05) for sheep supplied OEO compared with that for sheep supplied the CON (Table 3). Moreover, the protozoa abundance was greater (*P* < 0.05) for sheep supplied with CON at 4 h compared with that for sheep supplied OEO4 and OEO7 (Fig 3). This finding agreed with Ando et al. who reported that supplementing peppermint (200 g•d^-1^) to the ration fed to Holstein steers decreased the total protozoa concentrations [35]. Thao et al. who reported that supplementing eucalyptus EO (2 ml•hd^-1^•d^-1^) strongly inhibited the protozoa populations [14]. In agreement with these data, Patra et al. reported that the abundance of protozoa decreased linearly with increasing oregano oil doses in an *in vitro* test [6]. Moreover, Wanapat et al., Kongmun et al., and Kongmun et al. reported that supplementing beef cattle and buffalos with garlic powder resulted in lower protozoa numbers [36–38]. In contrast, several studies reported feeding 110 mg•d^-1^ mixed EO (thymol, guajacol, and limonene) to sheep resulted in no effects on rumen protozoa [39] and supplementing a steer ration with cinnamaldehyde oil (0.4–0.6 g•d^-1^) had no effect on total ruminal protozoa numbers [40]. The inconsistencies in the total ruminal protozoa populations reported in these studies were probably due to the differences in the chemical structures and properties of the different EO blends used. Each specific EO would have unique performance characteristics due to its chemical structure. In the rumen ecosystem, a large mount of H_2_ are produced in the hydrogenosomes of rumen protozoa [41], which will be used by hydrogenotrophic methanogens [42]. Actually, protozoa was demonstrated has ecto- and endo-symbiotic relationships with methanogenesis, and about 37% of rumen-derived CH_4_ could be produced by protozoa-associated methanogens [43, 44]. Therefore, feeding a specific EO that reduces ruminal protozoa populations could reduce the methane output by the rumen and increase energy availability to the animal [45, 46].

**Fig 4:**
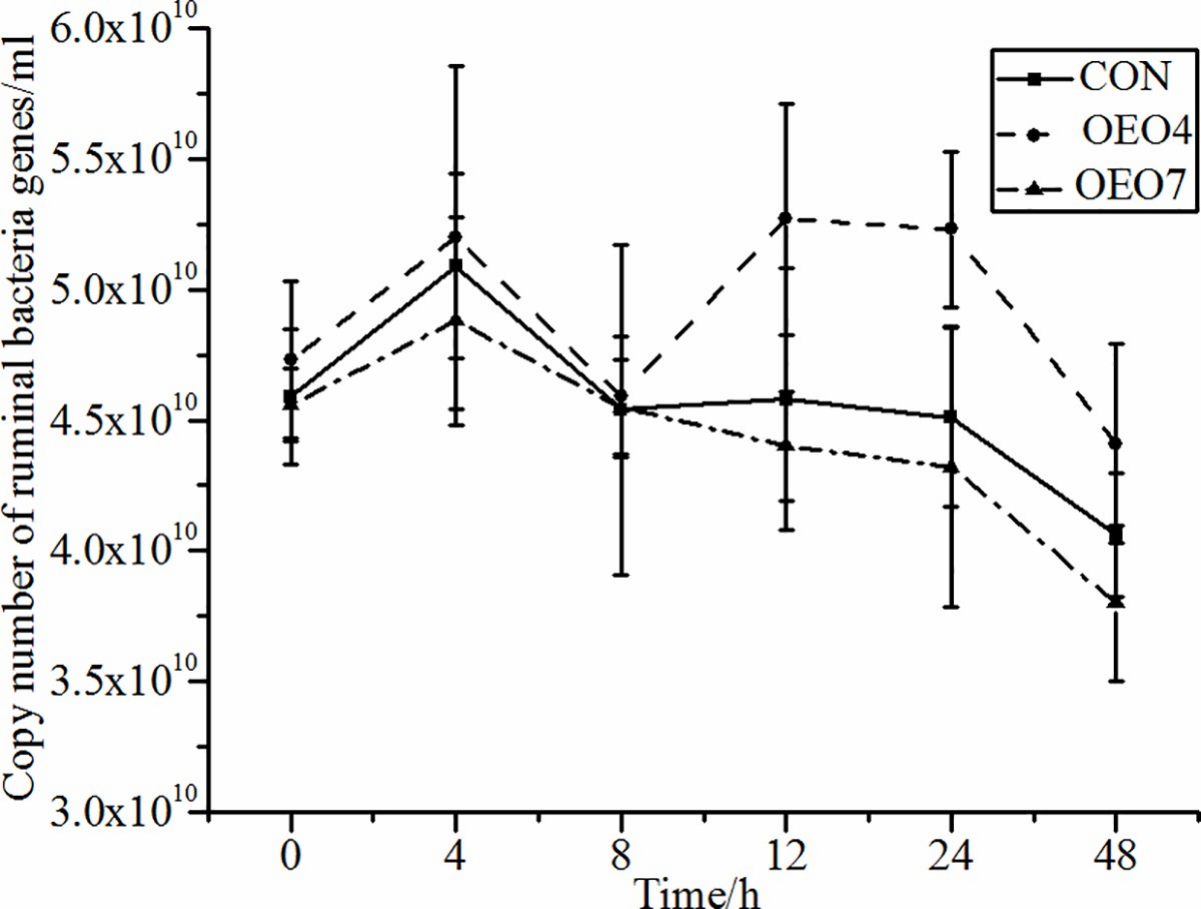
Abundance of ruminal Bacteria for sheep fed oregano essential oil (OEO) at 0 (CON), 4 (OEO4), or 7 (OEO7) g•d^-1^. ^a, b, c^ Means within the same collection time with unlike superscripts differ, (*P* < 0.05).

The total abundance of ruminal bacteria was similar (*P* = 0.080) between the sheep fed all treatment rations (Table 3), therefore supplementing OEO did not influence the abundance of ruminal bacteria. In accordance with previous studies, Benchaar et al. and Thao et al. reported that there was no change in the total bacterial population when sheep and swamp buffaloes were fed rations with mixed EO (thymol, eugenol, vanillin, and limonene) or eucalyptus EO (2 ml•hd^-1^•d^-1^) [47, 14]. While supplying OEO had no influence on the total abundance of bacteria (Fig 4), the sheep supplied OEO4 contained a greater (*P* < 0.05) abundance of *Ruminococcus flavefaciens* (*R*. *flavefaciens*), *Ruminococcus albus* (*R*. *albus*) and *Fibrobacter succinogenes* (*F*. *succinogenes*) than the sheep supplied CON and OEO7 (Table 3). As the representative cellulolytic bacterial species: these three species had a much higher cellulose digestibility than that of other cellulolytic ruminal species [29]. Our results corroborated of Wang et al., who reported that the NDF degradation rate was higher for sheep supplied 4 g•d^-1^ OEO (59.57%) than that for sheep supplied CON (46.26%) and 7 g•d^-1^ OEO (36.16%) [16]. Also, these results demonstrated that supplying a specific amount of EO (such as 4 g•d^-1^) could alter the microbial population of the rumen, but supplying a large amount of EO (such as 7 g•d^-1^) could have a detrimental impact on the microbial population. Carvacrol and thymol, as the main compounds of oregano oil, are phenolic structures that have more effective antimicrobials than other nonphenolic secondary plant metabolites due to the presence of a hydroxyl group in the phenolic structure [48–50]. The specific phenolic structure make them more attractive to the cell membrane structures, which could cause membrane expansion, increases fluidity and permeability, disturbs embedded proteins, inhibits respiration, and alters ion transport processes [51]. However, more studies are needed to deep understand the mechanisum of these active compounds and verify the result of this study.

**Fig 5:**
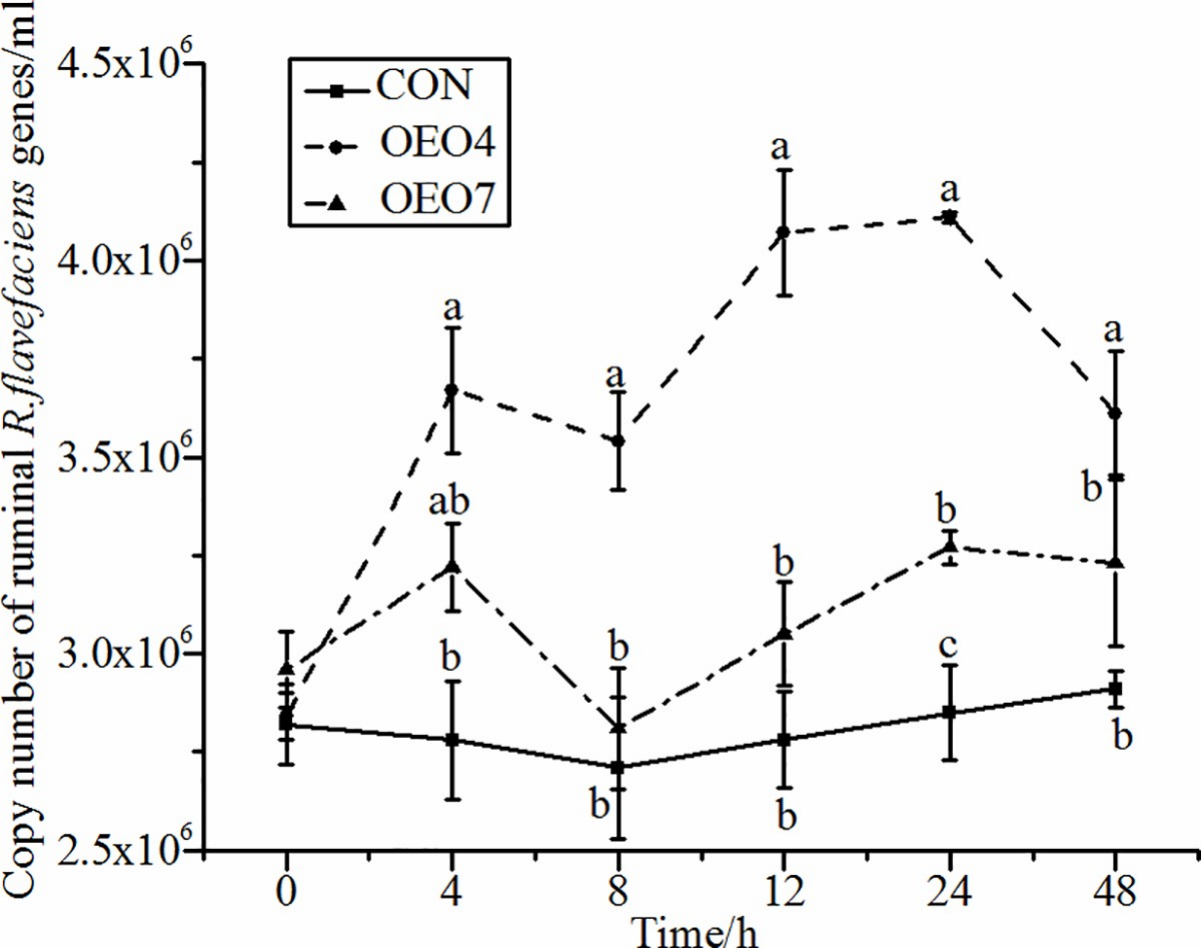
Abundance of ruminal *R*. *flavefaciens* for sheep fed oregano essential oil (OEO) at 0 (CON), 4 (OEO4), or 7 (OEO7) g•d^-1^. ^a, b, c^ Means within the same collection time with unlike superscripts differ, (*P* < 0.05).

**Fig 6:**
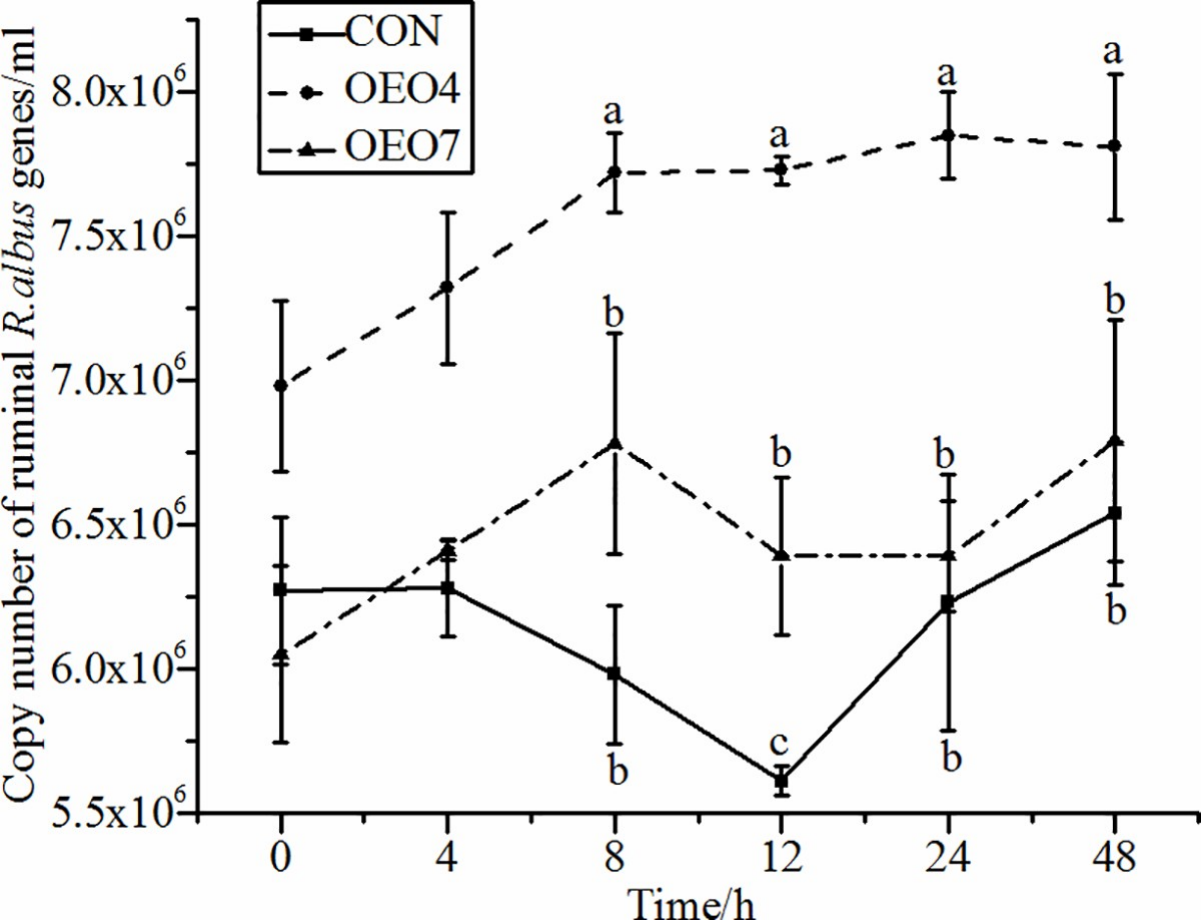
Abundance of ruminal *R*. *albus* for sheep fed oregano essential oil (OEO) at 0 (CON), 4 (OEO4), or 7 (OEO7) g•d^-1^. ^a, b, c^ Means within the same collection time with unlike superscripts differ, (*P* < 0.05).

**Fig 7:**
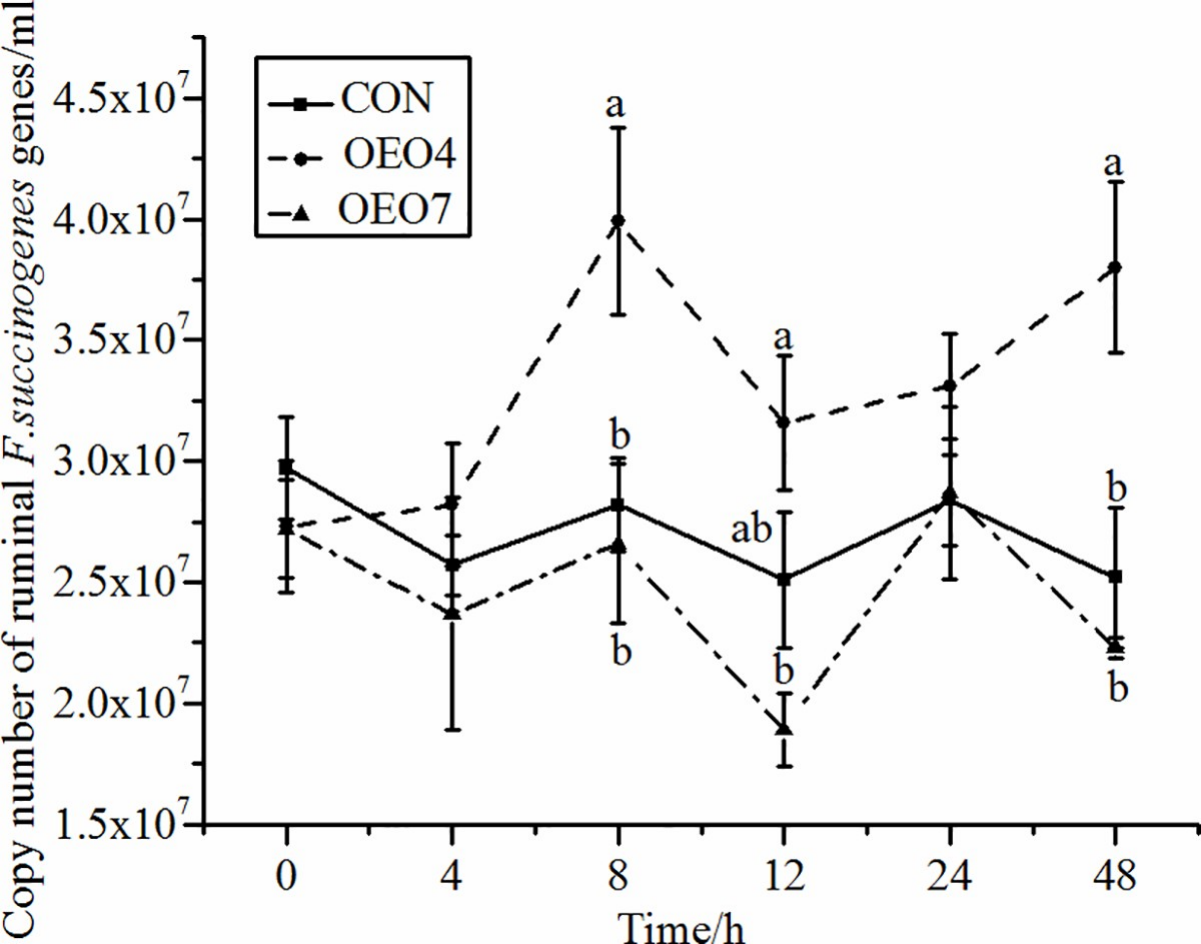
Abundance of ruminal *F*. *succinogenes* for sheep fed oregano essential oil (OEO) at 0 (CON), 4 (OEO4), or 7 (OEO7) g•d^-1^. ^a, b, c^ Means within the same collection time with unlike superscripts differ, (*P* < 0.05).

Supplying sheep with OEO resulted in significant (*P* < 0.05) changes in the abundance of *R*. *flavefaciens*, *R*. *albus*, and *F*. *sccinogenes* over time (Figs 5–7). The abundance of *R*. *flavefaciens* for sheep supplied OEO4 was greater (*P* < 0.05) than that for sheep supplied CON and OEO7 (Fig 5). Sheep supplied CON had the lowest abundance of *R*. *flavefaciens* compared with the sheep supplied OEO, indicating that supplying OEO to the sheep was benefit to the growth of *R*. *flavefaciens* (Fig 5). The abundance of *R*. *albus* increased (*P* < 0.05) for sheep fed OEO4 compared with that for sheep supplied CON and OEO7 8 h after supplying (Fig 6). The abundance of *F*. *succinogenes* was opposite that of *R*. *flavefaciens* and *R*. *albus* for sheep supplied OEO4, as it first decreased (*P <* 0.05) and then increased to a maximum at 8 h; it was the highest (*P <* 0.05) compared with that of sheep supplied CON and OEO7. These results indicated that the supplying rate of 4 g•d^-1^ of OEO favored the growth of *R*. *albus*, *R*. *flavefaciens*, *and F*. *succinogenes*, while the supplying rate of 7 g•d^-1^ could be detrimental to the growth of these microbes. Additionally, it could be explained by the findings of Wang et al., where the NH_3_-N concentrate was higher in sheep supplied OEO4 (16.78 mg•100 ml^-1^) compared with that in sheep supplied CON (14.38 mg•100 ml^-1^) and OEO7 (14.50 mg•100 ml^-1^) [16]. However, our results were in contrast to those of previous studies that demonstrated that fungal growth was inhibited by the presence of *Ruminococcus albus*, *Ruminococcus flavefaciens*, and *Butyrivibrio fibrisolvens* when the specimens were grown in coculture [52, 53].

Ruminal bacteria play a particularly critical role in the biological degradation of plant fibers [29]. In general, gram-positive bacteria were thought to be more sensitive to EO than gram-negative bacteria due to the lack of a protecting outer membrane surrounded the cell wall [8, 54]. However, not all researches on EOs have concluded that gram-negatives are less susceptible [55]. Patra and Yu did not observe a reduction in the population of *F*. *succinogenes* (gram-negative species) when supplementing with EOs, and these data are in agreement with our results [56]. It would be necessary to elucidate if the mechanism of action of EO would have inhibitory effects on other cellular structures because McIntosh et al. demonstrated that EO inhibited the growth of specific (i.e., *Clostridium sticklandii* and *Peptostreptococcus anaerobius*, both gram-positive bacteria) hyperammonia-producing (HAP) bacteria, but other HAP bacteria (i.e. *Clostridium aminophilum*) were less sensitive to the EO [37]. Wallace et al. who reported that the number of HAP bacteria was reduced by 77% in sheep receiving a low protein diet supplemented with mixed EOs (eugenol and limonene EO) at 100 mg•d^-1^ [57].

## Conclusions

The supplementation of oregano EO (4 g•d^-1^ or 7 g•d^-1^) had no effects on the ruminal pH and negative effects on the abundance of ruminal protozoa. In addition, supplementation with oregano EO increased the abundance of three primary cellulolytic bacteria (4 g•d^-1^) and the abundance of ruminal fungi (7 g•d^-1^). Our results suggest that supplying sheep with oregano EO could manipulate the rumen microbial. These results also demonstrate that adding a lower amount (such as 4 g•d^-1^) is beneficial to the ruminal microbial population, while adding higher (such as 7 g•d^-1^) amounts can be detrimental to the ruminal microbial population. However, the low number of animals used in this study, and the impacts of active components of oregano EO on rumen fermentation and the microbiota community also should be deepen studied in future.

## Supporting information

S1 File Minimal data of the paper. (PDF)
