# Supplementary material for: Effects of oregano essential oil on the ruminal pH and microbial population of sheep
Source: PLoS One. 2019 May 20;14(5):e0217054. doi: 10.1371/journal.pone.0217054 (PMC6527227; doi:10.1371/journal.pone.0217054)
Supplement: S1 File — (PDF) [file pone.0217054.s001.pdf]

Bacteria	CON	OE04	OE07
0.0000	11.1575	10.8075	10.5850
	10.6600	10.8800	10.4675
	10.0400	9.8125	10.6075
4.0000	10.6000	10.5600	10.2865
	10.2675	10.0500	10.6900
	10.3450	10.5550	10.4250
8.0000	10.8900	10.6575	10.6450
	10.6350	10.5700	10.4550
	10.2300	10.4167	10.5750
12.0000	10.7450	10.1350	10.5450
	10.6700	10.4275	10.7425
	10.2700	10.5125	10.5325
24.0000	10.7733	10.2703	10.3967
	10.3975	10.2950	10.5450
	10.5567	10.5275	11.0100
48.0000	10.7775	10.8500	10.6100
	10.8250	10.5500	10.8800
	10.5625	10.4275	10.9650
Bacteria	CON	OE04	OE07
0.0000	10.4720	10.5802	10.6489
	10.6258	10.5578	10.6853
	10.8174	10.8877	10.6420
	10.6384	10.6752	10.6587
4.0000	10.6443	10.6567	10.7412
	10.7471	10.8143	10.6165
	10.7231	10.6582	10.6984
	10.7048	10.7097	10.6854
8.0000	10.5547	10.6265	10.6304
	10.6335	10.6536	10.6891
	10.7587	10.7010	10.6520
	10.6489	10.6604	10.6572
12.0000	10.5995	10.7880	10.6613
	10.6227	10.6976	10.6003
	10.7463	10.6714	10.6652
	10.6562	10.7190	10.6423
24.0000	10.5907	10.7462	10.7071
	10.7069	10.7386	10.6613
	10.6577	10.6667	10.5176
	10.6518	10.7172	10.6287
48.0000	10.5895	10.5671	10.6412
	10.5748	10.6598	10.5578
	10.6559	10.6976	10.5315
	10.6067	10.6415	10.5768
Bacteria	CON	OE04	OE07
0.0000	2.9651E+10	3.8036E+10	4.4560E+10
	4.2245E+10	3.6123E+10	4.8446E+10

	6.5670E+10	7.7209E+10	4.3853E+10
	4.5855E+10	4.7339E+10	4.5576E+10
4.0000	4.4087E+10	4.5360E+10	5.5105E+10
	5.5855E+10	6.5204E+10	4.1353E+10
	5.2859E+10	4.5522E+10	4.9934E+10
	5.0934E+10	5.2029E+10	4.8797E+10
8.0000	3.5867E+10	4.2320E+10	4.2698E+10
	4.3003E+10	4.5039E+10	4.8879E+10
	5.7366E+10	5.0231E+10	4.4879E+10
	4.5412E+10	4.5863E+10	4.5485E+10
12.0000	3.9765E+10	6.1378E+10	4.5847E+10
	4.1945E+10	4.9845E+10	3.9836E+10
	5.5756E+10	4.6920E+10	4.6257E+10
	4.5822E+10	5.2714E+10	4.3980E+10
24.0000	3.8972E+10	5.5746E+10	5.0951E+10
	5.0920E+10	5.4773E+10	4.5847E+10
	4.5468E+10	4.6421E+10	3.2932E+10
	4.5120E+10	5.2313E+10	4.3243E+10
48.0000	3.8856E+10	3.6903E+10	4.3775E+10
	3.7565E+10	4.5684E+10	3.6123E+10
	4.5280E+10	4.9845E+10	3.4003E+10
	4.0567E+10	4.4144E+10	3.7967E+10
